# A Comparative Study of Radiofrequency Ablation, Microwave Ablation, and Percutaneous Ethanol Injection in Treatment of Hepatocellular Carcinoma—A Single-Center Experience

**DOI:** 10.3390/diagnostics15233027

**Published:** 2025-11-27

**Authors:** Mohamed Abdel-Samiee, Reham Reda Elkazaz, Hazem Omar, Nada Mohsen Salama, Asmaa Ibrahim Gomaa, Mohamed Akl Rady, Imam Waked

**Affiliations:** 1 Department of Hepatology and Gastroenterology, National Liver Institute, Menoufia University, Shebin El-Kom 32511, Egypt; 2 Department of Radiology, National Liver Institute, Menoufia University, Shebin El-Kom 32511, Egypt; 3 Radiology Department, National Cancer Institute, Cairo University, Cairo 11796, Egypt

**Keywords:** radiofrequency, ablation, microwave, percutaneous ethanol injection, hepatocellular carcinoma

## Abstract

**Background/Objectives**: Hepatocellular carcinoma (HCC) ranks as the third most prevalent cancer and is the second leading cause of cancer-related deaths globally. This study sought to evaluate microwave ablation (MWA), radiofrequency ablation (RFA), and percutaneous ethanol injection (PEI)—whether used separately or together (RFA+PEI, MWA+PEI)—for treating single HCC lesions ≤5 cm, focusing on outcomes, survival rates, complications, costs, and recurrence rates. **Methods**: This retrospective–prospective research study involved 250 patients with solitary HCC lesions measuring ≤5 cm, recruited from the National Liver Institute at Menoufia University. Patients were evenly divided into five groups, each containing (*n* = 50): RFA, MWA, PEI, combined RFA+PEI, and combined MWA+PEI. Indications and contraindications adhered to the Barcelona Clinic Liver Cancer (BCLC) guidelines. **Results**: Three patients were administered antiviral therapy 1–2 years after ablation. Average intervention costs were 17,340 ± 700, 31,200 ± 900, 1140 ± 300, 17,500 ± 0.0, and 33,800 ± 0.0 EGP for groups 1 through 5, respectively. Short-term advancement rates were 12%, 8%, 18%, 4%, and 2%. After 36 months, all patients survived six months after ablation. Average survival durations were 2.44 ± 1.17, 2.59 ± 1.02, 2.69 ± 0.99, 2.83 ± 1.06,and 2.91 ± 1.04 years, respectively. Complications were mainly minor (pain, nausea, and low-grade fever); one patient experienced an abscess and biloma post-MWA, one experienced minimal pleural effusion, and two combined-therapy cases had abdominal wall hematoma. **Conclusions**: RFA, MWA, and PEI—whether used individually or together—are successful treatment choices for early-stage HCC. The combination of MWA and PEI demonstrated the most favorable results, minimal recurrence rates, and the longest duration of progression-free survival.

## 1. Introduction

Hepatocellular carcinoma (HCC) ranks as the third most prevalent cancer globally and is responsible for the second highest rate of cancer-associated deaths [1]. The increasing occurrence of HCC is linked to various risk factors, mainly chronic infections with hepatitis B virus (HBV) and hepatitis C virus (HCV), along with alcohol-related liver disease, metabolic disorders, and, to a lesser extent, genetic conditions [2]. HCC may arise during earlier phases of metabolic dysfunction-associated steatotic liver disease (MASLD, formerly known as NAFLD) than in other chronic liver illnesses. A meta-analysis of 61 studies revealed that cirrhosis occurred in a smaller percentage of patients with MASLD-related HCC compared to those with HCC from alternative causes (61.5% versus 85.4%). The severity of fibrosis is a crucial factor in determining the risk of HCC. In a substantial United states of America (USA) cohort of 1773 MASLD patients, individuals with advanced F3 fibrosis demonstrated a greater incidence of HCC (0.34 per 100 person-years) compared to those with lower-stage fibrosis (F0–F2) or cirrhosis, although the HCC screening status remained uncertain [3]. Additional research indicates that MASLD patients lacking cirrhosis possess a significantly reduced risk of HCC compared to those with cirrhosis, although they frequently do not differentiate between F3 and earlier stages of fibrosis [4].

The management of patients with HCC is naturally complicated because of the frequent presence of chronic liver disease, the wide range of available diagnostic and treatment options, and the relative lack of strong scientific evidence supporting some commonly used methods. Consequently, physicians and allied health professionals, hospital administrators and policymakers, and patients themselves are often faced with difficult clinical and management choices [5].

Numerous studies have shown that the likelihood of developing de novo HCC remains even after attaining a sustained virological response (SVR) in individuals with advanced hepatic fibrosis (F3) but without cirrhosis. A meta-analysis of eight studies involving patients with F3 fibrosis found a combined incidence rate of HCC of0.5 per 100 person-years. Moreover, the incidence was comparable between those treated with interferon-based regimens (three studies; 0.4 per 100 person-years) and those treated with direct-acting antivirals (DAAs) (five studies; 0.5 per 100 person-years), suggesting that residual liver fibrosis rather than the antiviral regimen primarily drives the ongoing HCC risk [6,7].

In fact, various scores have been suggested to categorize HCC risk in patients with advanced chronic liver disease receiving DAAs [8,9]. In brief, factors such as failure to achieve SVR, cirrhosis, reduced albumin levels, elevated alpha-fetoprotein (AFP), low platelet count, and the presence of indeterminate liver nodules before starting DAA therapy are associated with an increased risk of HCC. Numerous factors indicate the severity of liver fibrosis and/or portal hypertension [10,11].

HCC can be diagnosed using noninvasive imaging criteria in at-risk populations or based on histopathological evidence in patients without known risk factors, and treatment selection is guided by the Barcelona Clinic Liver Cancer (BCLC) staging system [12]. In patients with advanced HCC, systemic treatments such as chemotherapy, targeted therapy, and immune checkpoint inhibitors have demonstrated significant improvements in progression-free survival and reductions in local recurrence rates [13].

Patients with early-stage disease, classified within the curative-intent category, are candidates for surgical resection or liver transplantation, which is considered the first-line option for those meeting the Milan criteria. However, the majority of patients are ineligible for these interventions due to impaired hepatic function or limited organ availability. Furthermore, postoperative hepatic dysfunction and high tumor recurrence rates remain major challenges. Consequently, radiofrequency ablation (RFA) and transarterial chemoembolization (TACE) have emerged as viable alternative therapeutic modalities [14].

For patients diagnosed with early-stage HCC, treatment options that can potentially cure include surgical resection, liver transplantation, and local ablation therapies. In contrast, transarterial therapies—such as TACE or radiotherapy—and systemic chemotherapy are generally considered palliative interventions [15].

RFA is a well-accepted alternative to surgical resection in treating hepatic tumors, especially HCC. This technique is regarded as both safe and effective, especially in patients with unresectable tumors due to multifocal disease or limited hepatic functional reserve resulting from liver cirrhosis [16,17].

Microwave ablation (MWA), another minimally invasive treatment modality, operates similarly to RFA. It employs imaging guidance ultrasound, computed tomography (CT), or magnetic resonance imaging (MRI) to accurately place a probe into the tumor. The probe emits microwaves to generate heat and destroy the malignant tissue. MWA is used for similar indications, such as RFA [18].

Percutaneous ethanol injection (PEI) has the longest track record among ablative therapies. It is relatively simple to perform, cost-effective, and requires minimal equipment. The therapeutic effect is achieved via direct injection of ethanol into the tumor, leading to cellular dehydration and ischemic necrosis via vascular thrombosis [19].

This study aimed to compare MWA, RFA, and PEI (either alone or in conjunction with RFA or MWA) for treating HCC by evaluating outcomes, survival, complications, costs, and tumor recurrence in a single HCC focal lesion measuring up to 5 cm.

## 2. Materials and Methods

### 2.1. Study Design and Population

This retrospective–prospective study involved 250 patients presenting with a single HCC lesion ≤ 5 cm in diameter (BCLC stage 0 or A). Other eligibility criteria, based on the BCLC guidelines for ablation therapy in HCC, included Child–Turcotte–Pugh (CTP) class A or early class B7 cirrhosis and non-resectable lesions. These patients were selected from 475 individuals deemed unfit for surgical intervention and referred to the hepatology and gastroenterology outpatient clinic or the Radiology Department of the National Liver Institute, Menoufia University. Key exclusion criteria included patients with CTP class C or advanced CTP class B8–B9 cirrhosis. Advanced or terminal-stage HCC were also excluded from our study. The study protocol was approved after local ethical committee approval by the Institutional Review Board of National Liver Institute (IRB number 00774/2025), Menoufia University. A written informed consent was obtained from all prospectively enrolled patients prior to inclusion in the study.

Liver cirrhosis (F4) was diagnosed when patients fulfilled more than one of these criteria: clinical and laboratory evidence of cirrhosis, ultrasonographic features suggestive of cirrhosis or noninvasive fibrosis scores (FIB-4 > 3.25 and APRI > 1.0). HCC was diagnosed according to updated EASL and AASLD guidelines using abdominal ultrasonography, triphasic CT, and dynamic MRI, interpreted by expert radiologists of the HCC Committee at the National Liver Institute, Shibin El-Kom, Egypt. Triphasic CT (arterial, porto-venous, and delayed phases) was performed using a Biograph multi-detector CT (Somatom biograph 128; Siemens Healthineers, Erlangen, Germany), and dynamic MRI was performed using a GE 1.5T scanner (Optima GE 450 W). Liver biopsy was performed when indicated [5,20].

The selected patients were categorized into five groups (50 patients per group) according to the therapeutic modality applied: group 1: RFA, group 2: MWA, group 3: PEI, group 4: combined radiofrequency ablation and percutaneous ethanol injection (RFA + PEI), and group 5: combined microwave ablation and percutaneous ethanol injection (MWA + PEI).

Each patient underwent a comprehensive pre-treatment evaluation, including recording data regarding age; gender; residence; past medical history; and baseline comorbidities, like the existence of diabetes mellitus, hypertension, and others. For identification of the cause of liver disease, HCV, HBV, autoimmune, cryptogenic, or metabolic causes were considered.

### 2.2. Clinical and Investigational Workup

Liver function assessment: total and direct bilirubin, aspartate aminotransferase (AST), alanine aminotransferase (ALT), and albumin were evaluated using the Cobas Integra 800 Auto Analyzer (Roche Diagnostics Ltd., Mannheim, Germany; Catalogue No. M-87432). Complete blood count (CBC): hemoglobin, white blood cell, and platelet counts were measured using the Sysmex KX-21 Analyzer (Sysmex Inc., Kobe, Japan). Renal function tests: serum urea and creatinine were assessed using the Cobas Integra 800 Auto Analyzer (Roche Diagnostics Ltd., Mannheim, Germany). Coagulation profile: prothrombin activity and international normalized ratio (INR) were measured using the BFT II Analyzer (Dade Behring, Marburg GmbH, Germany). Serum alpha-fetoprotein (AFP) was assessed using the Cobas Integra 800 Auto Analyzer (Roche Diagnostics Ltd., Mannheim, Germany; Catalogue No. M-87432).

Clinical scoring systems: the CTP score, Model for End-Stage Liver Disease (MELD) score, and BCLC staging system were used. Abdominal ultrasonography and triphasic CT scanning were performed for all patients. Dynamic magnetic resonance imaging (MRI) and/or liver biopsy were utilized when necessary to confirm the diagnosis.

#### 2.2.1. Treatment Allocation

Treatment selection was determined by a multidisciplinary team comprising a hepatologist, hepatic surgeon, and interventional radiologist. Local ablation therapy was indicated for patients with early-stage disease who were unsuitable for surgical resection or transplantation due to portal hypertension or comorbidities. The therapeutic modalities were categorized as follows:

* Radiofrequency Ablation (RFA):

Standard treatment for single HCC lesions ≤3 cm. Limitations included lesions adjacent to critical organs (e.g., kidney, colon, and gallbladder) or major vessels due to the heat-sink effect.

* Microwave Ablation (MWA):

Applied for single lesions 3–5 cm or those near the diaphragm, hepatic capsule, gallbladder, or major vessels. MWA is not influenced by the heat-sink effect and produces larger necrotic zones.

* Percutaneous Ethanol Injection (PEI):

Used for single HCC lesions ≤2 cm or for patients unable to afford other modalities due to its lower cost. Multiple sessions (once or twice weekly) were performed depending on tumor size, ethanol distribution, and patient condition. PEI was also preferred for lesions in high-risk locations where thermal ablation posed a risk of organ injury.

* Combined RFA + PEI:

Applied for single HCC lesions measuring 3–4 cm or located in high-risk areas (e.g., subcapsular, near major vessels >3 mm, or vital structures). The PEI needle was positioned closest to the vessel or organ at risk, while the RFA electrode was placed at least 10 mm away. Multiple overlapping ablations were performed to achieve an ablative margin ≥0.5 cm.

* Combined MWA + PEI:

Used for single HCC lesions 4–5 cm in size, particularly those adjacent to large vessels or in highly perfused regions. MWA provides rapid heating and greater necrosis, while PEI enhances treatment completeness.

Post-procedural care (following ablation): patients received adequate intravenous hydration (approximately 1000 mL of fluids), analgesics, antipyretics, antiemetics, prophylactic antibiotics, and liver-supportive therapy.

Careful observation of the puncture site for at least 6 h was performed with strict bed rest and avoidance of thigh flexion for 6 h, as well as monitoring of vital signs and peripheral pulses every 2 h for 24 h. All patients were discharged after a 24 h hospital stay.

All patients were evaluated three months post-ablation: this was performed using triphasic CT or MRI, liver function tests, and serum AFP measurement. Treatment response was classified based on imaging findings as: complete ablation (no enhancing tissue observed at the tumor site) or incomplete ablation (persistence of enhancing viable tumor tissue).

#### 2.2.2. Procedures

Radiofrequency Ablation Technique

Following skin disinfection with povidone iodine (Betadine), which also acted as a contact medium, local anesthesia was achieved by administering 10 mL of 2% lidocaine (Xylocaine; Astra) to numb the skin, subcutaneous tissue, muscles, and liver capsule along the predicted entry pathway. All RF ablations were conducted under real-time ultrasound guidance utilizing a 3.5 MHz probe with a free-hand method. It was applied to lesions found in the right lobe. An intercostal technique with the patient positioned in the left lateral decubitus stance was typically utilized for lesions in the left lobe; a subcostal technique was predominantly employed. The patients were conscious when the electrode was inserted into dome or left lobar lesions (sub-segment II).

During the procedure, vital signs including heart rate, blood pressure, respiration rate, and oxygen saturation were continuously monitored. The tumors’ dimensions were first assessed using standard ultrasound. We established a safety margin to cover the tumor along with a minimum of 5 mm of adjacent tissue.

Large tumors were ablated using several overlapping spheres. The sequence of ablation was based on the connection between the tumor and surrounding structures, as well as the blood supply. Once the tumor-feeding vessel was identified, we initially ablated the portion of the tumor where the vessel penetrated it.

For tumors located near significant structures like the diaphragm, gastrointestinal tract, or gallbladder, the protocol proved difficult to perform due to the restricted safety margin. An individualized protocol was created based on the tumor locations. The region of the tumor near the primary structure was deemed the important area. Appropriately handling the crucial zone to prevent harm to the nearby structure became the emphasis of the tailored plan. The RF ablation electrode was placed into the tumor at an angle perpendicular to the adjacent structure.

The radiofrequency systems used include internally cooled RF electrodes (RADIONICS, BURLINGTON, MASS).

Each RF energy application lasted 8–12 min; the RF electrodes were connected to a 500-kHz RF generator (model CC-1; Radionics) that could deliver 200 W of power. Throughout the process, a thermocouple integrated at the electrode tip constantly monitored the temperature of the surrounding tissue. Tissue impedance was observed through circuitry integrated into the generator. A peristaltic pump (Watson-Marlow, Medford, Mass) was used to deliver a 0 °C normal saline solution into the electrodes’ lumen at a flow rate adequate to maintain a tip temperature of 20–25 °C. No patient needed more than 3 L of saline solution. Grounding was accomplished by securing two dispersive pads, each exceeding 400 cm in surface area, to the patient’s thighs. In every instance, the complete treatment session lasted under 1 h.

Further imaging in the US was conducted following the conclusion of each treatment session. Alterations in the US during the ablation process were meticulously monitored for indications of intra-peritoneal bleeding.

Typically, patients were discharged 2–4 h following treatment if ultrasound showed no signs of active bleeding.

Microwave Ablation

MWA enables adaptable treatment options, such as percutaneous, laparoscopic, and open surgical access. Percutaneous microwave ablation is typically carried out with the patient under conscious sedation (obtained via intravenous delivery of midazolam and fentanyl, for instance), though in some cases where procedural pain is significant, general anesthesia might be necessary. Patients are monitored with continuous electrocardiography and pulse oximetry, along with blood pressure measurements taken every 5 min. Standard surgical preparation and draping are carried out. Local anesthetic is obtained through the injection of a 1% lidocaine hydrochloride solution both into the dermis and into deeper layers of tissue.

MWA devices consist of three main components: a generator, a flexible wire, and an antenna. The antenna utilized in MWA is commonly called a “needle” or other vague descriptors; however, the terms “applicator,” “antenna,” and “probe” are typically preferred for energy-based devices.

MWA is a thermal ablation technique that employs electromagnetic energy, leading to the spinning of water molecules. Various energy sources have been utilized to generate the required heat for initiating coagulation necrosis.

MWA generators presently support only two frequency bands: 915 MHz and 2.45 GHz.

Typically, heat is released in a centrifugal manner around the probe tip. Sufficient heat generated uniformly can effectively eliminate tumor cells near the antenna tip by denaturing intracellular proteins and cell membranes via the melting and dissolution of lipid bilayers. Depending on the energy delivery, ablation zones greater than 5 cm can also be attained. Intratumoral temperatures can be assessed using thermocouple probes that are placed separately or by utilizing the antenna itself (depending on the MW system). Coagulation occurs instantaneously at temperatures ranging from 60 to 100 °C, while vaporization and carbonization occur at temperatures exceeding 110 °C. The total ablation duration reached 300 s and persisted until the whole tumor was entirely enveloped by hyperechoic microbubbles on grayscale US.

During effective ablation, the temperature must be uniformly raised to approximately 50–60 °C for a minimum of 5 min. Elevated temperatures should be restricted to reduce the effects of vaporization and carbonization.

Vaporization refers to a phase change from the solid–liquid state to gas as vapor, observable in CT or ultrasound. Carbonization is defined as the transformation of a solid state into carbon, hindering the local spread of heat.

Percutaneous Ethanol Injection

The PEI procedure was carried out percutaneously under ultrasound guidance. We employed two or three needles to administer ethanol into multiple locations during a single procedure. Ethanol was injected twice a week. The process was carried out multiple times until it seemed that ethanol had been distributed throughout the tumor. To determine when to cease the repeated injection of ethanol and request a CT scan, we evaluated the total volume of ethanol injected and the alteration in echogenicity.

The standard guideline for the required volume of injected ethanol was determined using the following formula: V = (4/3) π (r + 0.5)^3^, where V (in milliliters) denotes the volume of ethanol, and r (in centimeters) represents the tumor’s radius; the addition of 0.5 accounts for a safety margin, based on the idea that adjacent liver parenchyma around the tumor, in addition to the tumor itself, should be ablated.

Radiofrequency Ablation and Percutaneous Ethanol Injection

RFA and PEI were conducted using a percutaneous method guided by real-time ultrasound (Aplio XV; Toshiba, Tokyo, Japan). Conscious sedation was achieved using pethidine and midazolam, with continuous monitoring of vital signs throughout the procedure. The tumor was first targeted by placing the RF electrode. The electrode was positioned 0.5 to 1 cm from the essential organs. PEI was conducted by injecting 1 to 10 mL of 99.5% ethanol using a 22-gauge percutaneous needle 15 to 20 cm long. The RFA program was launched right after PEI. Overlapping ablation was utilized to achieve an appropriate coagulation volume to encompass the whole tumor.

Microwave Ablation and Percutaneous Ethanol Injection

MWA and PEI were conducted using the percutaneous method with real-time ultrasound assistance. Conscious sedation was attained using pethidine and midazolam, along with monitoring of vital signs throughout the procedure. Dehydrated, sterile, 99.5% ethanol was administered slowly (1 mL per minute) into HCCs, while multipoint revolving insertion occurred using one or two 21-gauge percutaneous transhepatic cholangiography (PTC) needles. Simultaneously, microwave radiation expanded the coagulation area near the gallbladder through the spread of ethanol. The amount of ethanol delivered was influenced by the tumor’s size and position. For extensive regions of the tumor near the gallbladder that underwent inadequate ablation, we delivered a greater volume of ethanol.

The unablated tumor volume was assessed by measuring the width and the length from the ablation margin to the gallbladder. The same volume of injected ethanol was initially determined prior to treatment and modified during the operation based on the extent of the unablated tumor. The spread of ethanol was additionally observed using grayscale US, and we refrained from injecting ethanol into blood vessels or the gallbladder. During the withdrawal of the antenna, the needle pathway was clotted to prevent bleeding and tumor cell seeding.

#### 2.2.3. Patient Preparation

Patients fasted overnight and were admitted to the hospital on the morning of the procedure.

Prophylactic antibiotics (1 g of cefazolin and 500 mg of metronidazole) and an antiemetic (24 mg of ondansetron hydrochloride) were administered.

#### 2.2.4. Statistical Analysis

To analyze our study data, we used SPSS version 25.0 (IBM, SPSS, Statistics for Windows, IBM Corp., Armonk, NY, USA). Mean ± SD was used for quantitative variables, and frequency and percentage were used for qualitative variables. ANOVA was employed in multiple groups with normal variables, while the Kruskal–Wallis test was used for non-normal variables to assess group differences. Chi-square analysis was used to assess differences in the frequency of qualitative variables. The statistical methods assumed a significance value of *p* < 0.05.

## 3. Results

The baseline comparative analysis for demographic data was illustrated in Table 1. There were no statistically significant differences for all baseline parameters, while there was a statistically significant difference for liver cirrhosis, HCV, and bilharziasis.

Table 2 showed that the mean ± SD of tumor diameter in all studied groups was (2.7 ± 0.86; 3.73 ± 0.53; 2.02 ± 0.54; 3.59 ± 0.41; 4.05 ± 0.47, respectively).

Table 3 indicated the analysis of all studied patients according to the number of therapeutic sessions required. In the RFA group, a single session was adequate for 44 patients (88%), whereas 6 patients (12%) required two sessions. All patients in the MWA group (100%) achieved complete ablation following a single session. In contrast, among patients treated with PEI, four sessions were sufficient for 46 patients (94%), while six sessions were required for 4 patients (6%). For the combined modality groups, a single session of RFA with PEI and a single session of MWA with PEI were adequate for all patients (100% in each group).

**Figure 1:**
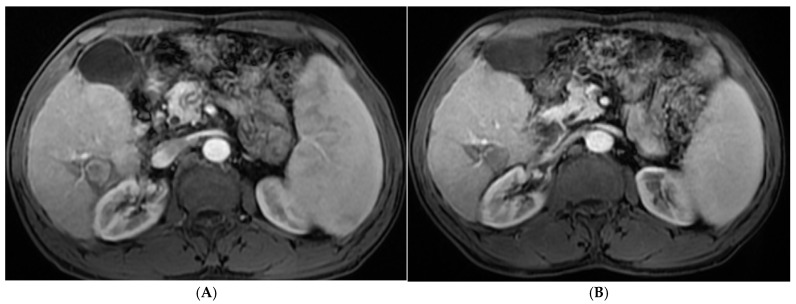
(**A**). Triphasic MRI abdomen (arterial phase) axial cuts revealing right lobe segment VI focal lesion with homogeneous enhancement (before RFA). (**B**). The same lesion (arterial phase) axial cuts one month after RFA.

**Figure 2:**
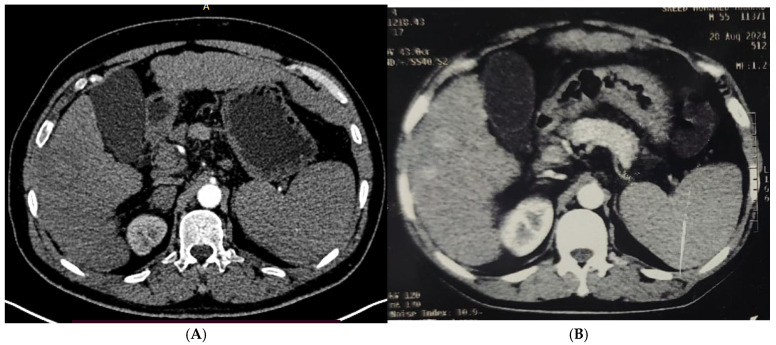
(**A**). Triphasic CT abdomen (arterial phase) axial cuts revealing, in right lobe segment V, two adjacent focal lesions with homogeneous enhancement (before MWA). (**B**). The same lesion (arterial phase) axial cuts one month after MWA.

**Figure 3:**
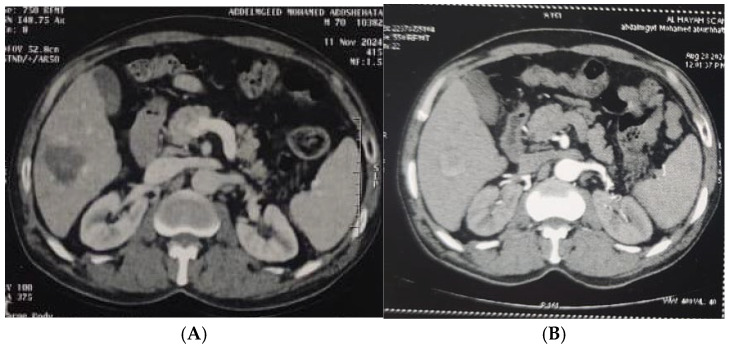
(**A**). Triphasic CT abdomen (arterial phase) axial cuts revealing right lobe segment VI focal lesion with homogeneous enhancement (before MWA). (**B**). The same lesion (arterial phase) axial cuts one month after MWA.

**Figure 4:**
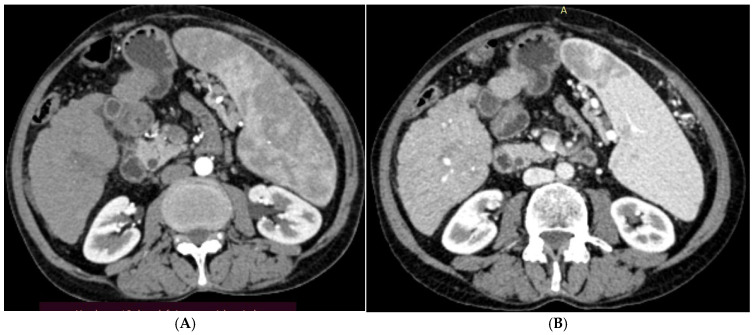
(**A**). Triphasic CT abdomen (arterial phase) axial cuts revealing right lobe segment VI focal lesion with homogeneous faint enhancement (before ethanol injection). (**B**). The same lesion (arterial phase) axial cuts one month after EI.

Table 4 and Figure 1, Figure 2, Figure 3 and Figure 4 illustrated a statistically significant difference in the response to intervention among all studied patients classified according to outcome (complete or incomplete response) three months post-intervention across groups (1–5). Complete response was achieved by 46 patients (92%), 47 patients (94%), 45 patients (90%), 49 patients (98%), and 50 patients (100%) in groups 1 through 5, respectively. Conversely, four patients (8%), three patients (6%), five patients (10%), one patient (2%), and zero patients (0%) in the corresponding groups exhibited an incomplete response.

Table 5 showed a statistically significant difference among all studied patients according to tumor recurrence (no recurrence, local, distant, or both local and distant): in group 1, 41 patients (78%) showed no recurrence, 4 patients (8%) developed local recurrence, 3 patients (6%) had distant recurrence, and 2 patients (4%) exhibited both local and distant recurrence. In group 2, 44 patients (88%) had no recurrence, 4 patients (8%) developed local recurrence, 2 patients (4%) had distant recurrence, and 2 patients (4%) experienced both local and distant recurrence. In group 3, 34 patients (60%) showed no recurrence, 10 patients (20%) developed local recurrence, and 6 patients (12%) had distant recurrence. In group 4, 46 patients (92%) had no recurrence, 2 patients (4%) developed local recurrence, 1 patient (2%) had distant recurrence, and 1 patient (2%) developed both local and distant recurrence, while in group 5, 49 patients (96%) showed no recurrence, and 2 patients (4%) developed distant recurrence.

Recurrence patterns varied among the five study groups and were clearly described in Table 6. In group 1, most recurrences occurred at 12 months (66.7%), with fewer cases at 6 months (11.1%) and 18 months (22.2%). Group 2 had the highest recurrence at 12 months (66.7%), with isolated cases at 18 and 24 months (16.7% each). In group 3, recurrences were distributed at 6 months (37.5%), 12 months (25.0%), and 24 months (37.5%). Group 4 experienced recurrences predominantly at 12 months (50.0%), with smaller proportions at 18 and 24 months (25.0% each). In group 5, recurrences occurred equally at 18 and 24 months (50.0% each). Overall, early recurrences (6–12 months) were common, while later recurrences (18–24 months) were less frequent and variable.

During the five-year follow-up, 250 patients survived at least six months after ablation. The mean survival time from six months to five years showed a gradual increase across groups (group 1: 2.44 ± 1.1, group 2: 2.59 ± 1.02, group 3: 2.69 ± 0.99, group 4: 2.83 ± 1.06, group 5: 2.91 ± 1.04). Overall survival was high, with 80% of patients in group 1 and 88–90% in groups 2–5 remaining alive at the end of the study. Mortality was low, ranging from 10% to 20%, and only a single patient in group 3 had an unknown status. These findings in Table 7 indicate a trend toward improved survival in the higher-numbered groups, with consistently favorable long-term outcomes across all groups.

## 4. Discussion

HCC is the most prevalent primary malignant tumor in the liver. The occurrence of HCC is rising globally, leading to approximately 690,000 deaths annually, making it the third leading cause of cancer-related mortality [21]. HCC poses a significant worldwide health issue because of its increasing prevalence and unfavorable outcome [5].

RFA, first introduced by Rossi et al. in 1993, induces coagulative necrosis through ionic agitation and subsequent tissue heating. Advances in RFA technology have rendered its outcomes comparable to surgical resection in early-stage BCLC disease, particularly when considering operative duration, intraoperative bleeding, and disease-free survival. Despite certain limitations, RFA remains the curative standard for HCC lesions smaller than 3 cm in diameter [22].

PEI represents another ablative approach that mitigates the heat-sink effect by promoting coagulation and occluding small intratumoral vessels. Although PEI alone has demonstrated inferior efficacy compared with RFA across tumor sizes, pre-treatment with PEI prior to RFA may theoretically enhance ablation efficiency by overcoming the heat-sink effect, particularly in anatomically challenging hepatic regions [23].

Meta-analyses by Zhu et al. (2016) and Luo et al. (2017) demonstrated that RFA-PEI significantly improved overall survival and reduced local recurrence compared with RFA alone, albeit with a higher incidence of fever [7,24]. However, both studies were limited by small sample sizes and the absence of subgroup analyses stratified by tumor location, study design, or needle type [25,26].

The proposed mechanisms underlying the synergistic effect of combined (RFA-PEI) include (1) enhanced ethanol ablation due to its low boiling point (78.3 °C), (2) embolization of microvasculature to minimize heat dissipation, (3) diffusion of ethanol into regions inaccessible to RFA, (4) extension of the ablation zone to ensure a wider safety margin, and (5) reduced tissue carbonization, thereby facilitating improved thermal conduction. These mechanisms support the rationale for integrating local–regional therapies to optimize HCC treatment outcomes [23].

Our study was a retrospective–prospective study carried out on 250 patients who attended the HCC clinic at the National Liver Institute and underwent RFA, MWA, PEI, combination RFA and PEI, or MWA and PEI.

The present study was intended to evaluate RFA, MWA, PEI, RFA and PEI, and MWA and PEI for HCC patients as first-line treatments according to response to ablation, recurrence, complications, cost, and overall survival.

Patients were followed up from the time of enrollment to the date of death or date of data collection if they remained alive, with a minimum follow-up period of 1 year and a maximum period of 5 years.

In our study, RFA was performed on a single HCC focal lesion with a mean diameter of 2.70 ± 0.56 cm. A complete radiological response was achieved in 92% of patients, and an incomplete response was observed in 8%. This is very close to the results published by N’Kontchou et al. (2009), who reported that complete radiological ablation was accomplished in 222 patients (94.7%), while treatment failure was noted in 13 out of 307 nodules following RFA [10,27].

In our study, after RFA, recurrence developed in 18% of patients at 6, 12, and 18 months, with rates of 11.1%, 66.7%, and 22.2%, respectively. Local recurrence was reported in 8% of patients, distant recurrence in 6%, and local and distant recurrence in 4%. Complications (4%) included minimal pleural effusion and abdominal wall hematoma.

In our study, MWA was performed on HCC with a mean tumor diameter of 3.73 ± 0.53 cm. A complete radiological response was achieved in 94% of patients, and an incomplete response was observed in 6%. This is very close to the results published in Dong et al., 2014 [3]. Microwave coagulation therapy (MCT) can achieve complete tumor ablation in 89–94% of cases [28].

In our study, after MWA, recurrence developed in 12% of tumors after 12, 18, and 24 months in 66.6%, 16.7%, and 16.7% of patients, respectively. Local recurrence occurred in 8% of patients, distant recurrence in 4%, and complications in 4%, including liver abscess and biloma.

In a retrospective study, Ai-Xue Sun et al. (2015) examined 182 patients with one medium-sized HCC who received percutaneous MWA [29]. The projected rate of technical effectiveness was 93%. The rate of major complications, which included liver abscess in four patients, was 2.7%, with one patient experiencing abdominal bleeding at the puncture site. In their research, one patient passed away due to septicemia linked to a liver abscess (the 30-day mortality rate was 0.5%) [30].

A retrospective study conducted by Xue et al. (2021) assessed and compared the therapeutic outcomes of MWA in 53 patients with HCCs [16]. Complete local tumor control was observed in 84.4% of lesions treated with RFA and in 88.9% of lesions treated with MWA. Nonetheless, in both groups, technical success was attained in lesions smaller than 2 cm. The recurrence rates were 6.3%, 3.1%, 3.1%, and 3.1% at 3, 6, 9, and 12 months for RFA compared with 0%, 5.6%, 2.8%, and 2.8% for MWA, respectively. Local progression rates were 96.9%, 93.8%, and 90.6% at 1, 2, and 3 years, respectively, for RFA-treated patients, whereas MWA-treated patients had rates of 97.2%, 94.5%, and 91.7%, respectively [31].

In our study, according to PEI, performed in patients with HCC and a mean tumor diameter of 1.82 ± 0.54 cm, a complete radiological response was achieved in 90% of patients, and an incomplete response was observed in 10%. Local recurrence was achieved in 20% of patients, and distant recurrence was achieved in 18% of patients within 6, 12, 18 months, 37.5%, 25%, and 37.5%, respectively. No complications occurred. A cohort study demonstrated that complete ablation by PEI was reached in over 90% of instances, with a local recurrence rate below 1% [32].

In our study, RFA and PEI were performed on HCC with a mean tumor diameter of 3.59 ± 0.41 cm. A complete radiological response was achieved in 98% of patients, an incomplete response was observed in 4%, and recurrence was achieved in 8% of patients, with a local recurrence rate of 4%, a distant recurrence rate of 2%, and a local and distant recurrence rate of 2%. This is very close to the result published by Kalra (2017), where fifty patients showed that eight and six patients treated with RFA and alcohol showed local and distant intrahepatic tumor recurrence, respectively [5]. Every local recurrence occurred within 1.5 to 15 months during the observation period. The distant intrahepatic recurrences took place at 6–72 months throughout the follow-up period [24].

In our study, MWA and PEI were performed on HCC with a mean tumor diameter of 4.05 ± 0.47 cm. A complete radiological response was achieved in 1 00% of patients. Distant recurrence developed in 4% of patients within 18 to 24 months at a rate of 50% and 50%, respectively, with 2% experiencing complications, such as abdominal wall hematoma.

A study by Vogl et al. (2017) found that the combination of PEI and MW ablation resulted in a significantly wider maximum diameter of coagulation and more complete necrosis of the tumors that were treated [15]. This was considered to result from enhanced thermal conduction and diffusion in tissue that had been previously coagulated [33].

In our study, overall survival was determined: the 1-year, 3-year, and 5-year overall survival for the 250 patients was 92.4%, 66.5%, and 38.4%, respectively, with a median survival duration of 2.5 years. Another study showed that the estimated 1-, 3-, 5-, and 10-year overall survival rates were 90.0%, 70.8%, 41.1%, and 28.4%, respectively [34].

Iterative RFA, MWA, PEI, or a mix of RF with PEI, or MWA with PEI, continues to be a safe and highly effective option in certain instances of restricted recurrence. The necessity for surgical interventions through resection was relatively minimal (just three patients).

In 14 patients with multiple nodular recurrences and 7 patients with a partial response, TACE was indicated in patients with adequately preserved liver function. The other three patients experiencing advanced recurrence were given supportive treatment.

## 5. Conclusions

As a major contributor to cancer-related deaths, HCC presents a considerable global health issue that requires tailored strategies for various social and healthcare settings. RFA, MWA, and PEI, whether used individually or together, are viewed as potential curative treatments for patients with very early or early HCC, demonstrating favorable results in overall survival, recurrence-free survival, and progression-free survival. Combination therapy yields superior outcomes compared to monotherapy regarding tumor size; specifically, combination MWA and PEI is more effective for a single HCC focal lesion 4–5 cm, RFA and PEI are preferable for a single HCC focal lesion 3–4 cm, MWA is optimal for a single HCC focal lesion 2–3 cm, and RFA is best for treating a single HCC ≤ 3 cm. Combination therapy is more effective in high-risk areas of HCC, leading to improved results in terms of complications, outcomes, and recurrence.

## Figures and Tables

**Table 1:** Comparison between the different studied groups according to demographic data.

Demographic Data	RFA (*n* = 50)		MW (*n* = 50)		PEI (*n* = 50)		RFA&PEI (*n* = 50)		MW& PEI (*n* = 50)		Test of Sig. (*p*)
	No.	%	No.	%	No.	%	No.	%	No.	%	
Gender											
Male	38	76.0	37	74.0	42	84.0	39	78.0	38	76.0	**χ^2^ =** 1.454 (0.835)
Female	12	24.0	13	26.0	8	16.0	11	22.0	12	24.0	
History of hypertension											
Negative	37	74.0	38	76.0	39	78.0	36	72.0	40	80.0	1.096(0.895)
Positive	13	26.0	12	24.0	11	22.0	14	28.0	10	20.0	
History of diabetes mellitus											
Negative	36	72.0	34	68.0	34	68.0	37	74.0	35	70.0	0.653 (0.957)
Positive	14	28.0	16	32.0	16	32.0	13	26.0	15	30.0	
Bilharziasis											
Negative	29a	58.0	25	50.0	31	62.0	39	78.0	39	78.0	13.680 * (0.008 *)
Positive	21a	42.0	25	50.0	19	38.0	11	22.0	11	22.0	
HBV											
Negative	47	94.0	46	92.0	49	98.0	44	88.0	48	96.0	4.564 (MCp = 0.343)
Positive	3	6.0	4	8.0	1	2.0	6	12.0	2	4.0	
HCV											
Negative	9a	18.0	21b	42.0	10a	20.0	20b	40.0	12ab	24.0	12.601 * (0.013 *)
Positive	41a	82.0	29b	58.0	40a	80.0	30b	60.0	38ab	76.0	
Liver cirrhosis											
No	1	2.0	0	0.0	0	0.0	0	0.0	0	0.0	317.809 * (<0.001 *)
Yes	49	98.0	50	100.0	50	100.0	50	100.0	50	100.0	
Performance status											
0	49	98.0	48	96.0	46	92.0	47	94.0	47	94.0	2.196 (0.821)
1	1	2.0	2	4.0	4	8.0	3	6.0	3	6.0	
Child–Turcotte–Pugh score (CTP)											
A5	21	42.0	16	32.0	21	42.0	14	28.0	14	28.0	χ^2^ = 6.269 (0.617)
A6	20	40.0	26	52.0	20	40.0	28	56.0	28	56.0	
B7	9	18.0	8	16.0	9	18.0	8	16.0	8	16.0	
Age											
Min.–Max.	33.0–74.0		40.0–73.0		48.0–78.0		48.0–75.0		45.0–70.0		F = 0.341 (0.850)
Mean ± SD.	59.72 ± 8.12		60.22 ± 7.23		60.10 ± 7.32		60.30 ± 7.24		61.34 ± 6.53		
Median	62.50		60.0		61.50		62.0		64.0		

χ^2^:Chi-square test. F: ANOVA test. MC: Monte Carlo. *p*:*p* value for comparing between the studied groups. *: Statistically significant at *p* ≤ 0.05.

**Table 2:** Comparison between the different studied groups according to the size of focal lesion.

	RFA (*n* = 50)	MW (*n* = 50)	PEI (*n* = 50)	RFA&PEI (*n* = 50)	MW&PEI (*n* = 50)	H (*p*)	
Largest tumor diameter							
Min.–Max.	2.0–3.50	3.0–5.0	1.0–2.50	3.0–4.50	4.0–5.0	155.389 * (<0.001 *)	
Mean ± SD.	2.70 ± 0.56	3.73 ± 0.53	1.82 ± 0.54	3.59 ± 0.41	4.50 ± 0.47		
Median	2.80	3.50	2.0	3.50	4.0		
Significance between groups		*p*_1_ < 0.001 *, *p*_2_ = 0.002 *, *p*_3_ < 0.001 *, *p*_4_ < 0.001 *, *p*_5_ < 0.001 *, *p*_6_ = 0.570, *p*_7_ = 0.013 *, *p*_8_ < 0.001 *, *p*_9_ < 0.001 *, *p*_10_ = 0.002 *					

**Kruskal–Wallis test** pairwise comparison between Each of the 2 groups completed a **Post Hoc Test (Dunn’s multiple comparisons test)**. *p*:*p* value for comparing between the studied groups. *p*_1_: *p* value for comparing between **RFA** and **MW**. *p*_2_: *p* value for comparing between **RFA** and **PEI**. *p*_3_: *p* value for comparing between **RFA** and **RFA&PEI**. *p*_4_: *p* value for comparing between **RFA** and **MW&PEI**. *p*_5_: *p* value for comparing between **MW** and **PEI**. *p*_6_: *p* value for comparing between **MW** and **RFA&PEI**. *p*_7_: *p* value for comparing between **MW** and **MW&PEI**. *p*_8_: *p* value for comparing between **PEI** and **RFA&PEI**. *p*_9_: *p* value for comparing between **PEI** and **MW&PEI**. *p*_10_: *p* value for comparing between **RFA&PEI** and **MW&PEI**. *: Statistically significant at *p* ≤ 0.05.

**Table 3:** Distribution of the studied cases according to number of treatment sessions.

No. of Sessions	RFA (*n* = 50)		MWA (*n* = 50)		PEI (*n* = 50)		RFA&PEI (*n* = 50)		MW&PEI (*n* = 50)	
One session	44	88.0%	50	100%	0	0.0%	50	100%	50	100%
Two sessions	6	12.0%	0	0.0%	0	0.0%	0	0.0%	0	0.0%
Four sessions	0	0.0%	0	0.0%	46	92.0%	0	0.0%	0	0.0%
Six sessions	0	0.0%	0	0.0%	4	8.0%	0	0.0%	0	0.0%

**Table 4:** Comparison between the different studied groups according to response to intervention after 3 months.

	RFA (*n* = 50)		MW (*n* = 50)		PEI (*n* = 50)		RFA&PEI (*n* = 50)		MW& PEI (*n* = 50)		χ^2^(*p*)
	No.	%	No.	%	No.	%	No.	%	No.	%	
Incomplete response	4	8.0	3	6.0	5	10.0	1	2.0	0	0.0	33.837 * (<0.001 *)
Complete response	46	92.0	47	94.0	45	90.0	49	98.0	50	100.0	

χ^2^: Chi-square test. *p*:*p* value for comparing between the studied groups. *: Statistically significant at *p* ≤ 0.05.

**Table 5:** Comparison between the different studied patients regarding recurrence.

	RFA (*n* = 50)		MW (*n* = 50)		PEI (*n* = 50)		RFA&PEI (*n* = 50)		MW& PEI (*n* = 50)		χ^2^(*p*)
	No.	%	No.	%	No.	%	No.	%	No.	%	
No recurrence	41	78.0	44	88.0	34	60	46	92.0	48	96.0	33.837 * (<0.001 *)
Local recurrence Distant recurrence	4 3	8.0 6.0	4 2	8.0 4.0	10 6	20.0 8.0	2 1	4.0 2.0	02	0.0 4.0	
Local and distal recurrence	2	4.0	0	0.0	0	0.0	1	2.0	0	0.0	

χ^2^: Chi-square test. *p*: *p* value for comparing between the studied groups. *: Statistically significant at *p* ≤ 0.05.

**Table 6:** Time of recurrence among the study groups.

Recurrence Time/Months	RFA (*n* = 50)		MW (*n* = 50)		PEI (*n* = 50)		RFA&PEI (*n* = 50)		MW&PEI (*n* = 50)		χ^2^(*p*)
	No.	%	No.	%	No.	%	No.	%	No.	%	
	**(*n* = 9)**		**(*n* = 6)**		**(*n* = 16)**		**(*n* = 4)**		**(*n* = 2)**		11.563 (^MC^ *p* = 0.365)
6 moths	1	11.1	0	0.0	6	37.5	0	0.0	0	0.0	
12 months	6	66.7	4	66.6	4	25.0	2	50.0	0	0.0	
18 months	2	22.2	1	16.7	6	37.5	1	25.0	1	50.0	
24 months	0	0.0	1	16.7	0	0.0	1	25.0	1	50.0	

χ^2^:Chi-square test. *p*: *p* value for comparing between the studied groups.

**Table 7:** Descriptive analysis of the studied cases according to survival time (*n* = 250).

	Min.–Max.	Mean ± SD.		Median
**Survival time** (in years)	0.6–5.0	2.71 ± 1.06		2.50
Survival time	**No.**		**%**	
1 year	231		92.4	
3 years	125		66.5	
5 years	96		38.4	

## Data Availability

All data are available upon request from the corresponding author due to ethical constriction.
